# Key focal areas for bridging the fields of aging and disability: findings from the growing older with a disability conference

**DOI:** 10.5334/ijic.1082

**Published:** 2012-11-16

**Authors:** Vishaya Naidoo, Michelle Putnam, Andria Spindel

**Affiliations:** York University, Toronto, Ontario, Canada; Simmons College School of Social Work, Boston, Massachusetts, USA; March of Dimes Canada, Toronto, Ontario, Canada

**Keywords:** Aging, disability, bridging, knowledge translation, aging with disability, policy

## Abstract

Based upon research presented at the 2011 Festival of International Conferences on Caregiving, Disability, Aging and Technology (FICCDAT)—and specifically the Growing Older with a Disability (GOWD) conference, this paper identifies areas where bridging building between aging and disability is needed to support older adults aging into or with disabilities. Five focal areas emerged: 1) The Need to Forward Bridging Between Aging and Disability Sectors, 2) Theoretical Frameworks of Individual Aging that Facilitate Bridging, 3) Bridging through Consumer Participation and Involvement, 4) Bridging Through Knowledge Transfer and 5) Bridging Opportunities in Long-Term Supports and Services and Assistive Technologies. Discussion of themes is provided within both international and Canadian contexts, reflecting the interests of FICCDAT and GOWD organizers in discussing how to improve bridging in Canada. Findings from this report form the basis of the Toronto Declaration on Bridging Aging and Disability Policy, Practice, and Research.

## Introduction

In recognition of the importance of ideas presented at the FICCDAT meeting, the Office for Disability Issues, Government of Canada commissioned a report on conference findings that was led by the March of Dimes Canada (Toronto, ON), a principal sponsor of the Growing Older with a Disability (GOWD) conference at the 2011 Festival of International Conferences on Caregiving, Disability, Aging, and Technology (FICCDAT) [1]. The report was based upon the proceedings of GOWD and aimed to establish the principles needed to support older adults aging into or with disabilities and to bridge knowledge and practice in aging and disability. This report had three phases of development. In the first phase, a purposeful sample of scholarly papers presented at GOWD were reviewed in order to identify the scope of issues analyzed in the presentations and specific themes related to the support of older adults aging with or into disability. In the second phase, scholarly literature related to key concepts that emerged at GOWD was reviewed. In the third and final phase, the panel of experts who wrote the Toronto Declaration was consulted in a peer-review process to assess the findings of this paper. This is an abbreviated version of that report, highlighting the key focal areas for activities related to bridging that emerged at GOWD. This abbreviated report supplements the editorials in this supplemental issue and references the editorials, the Toronto Declaration, and a selected set of GOWD conference presentations.

### GOWD 2011: Working to bridge aging and disability

The GOWD 2011 meeting was timely, in that trends in global aging and disability are pushing policy-makers internationally to confront the realities of aging populations. GOWD 2011 presentations highlighted the substantial shared needs and concerns of persons growing older with disability and those growing older into disability, making the case for moving efforts to bridge the fields of aging and disability forward. This review places the findings from GOWD into five Key Focal Areas for bridging work with the aim of contributing to research, practice, and policy agendas attempting to bridge aging and disability.

### Distinguishing between aging into disability versus aging with disability at GOWD

The GOWD 2011 meeting considered issues of both aging with and aging into disability. Aging *into* disability refers to people who acquire a disability in the later years of life for the first time and who otherwise lived without impairment in childhood or middle age. This process can occur in one of two ways. The first is through the sudden onset of a disability-causing condition in old age—such as hearing loss or reduced cognitive function after a stroke—while the second occurs through slower advancement of symptoms of functional or cognitive decline that may result from other health conditions [2]. The experience of aging *with* a disability is a newer concept, referring to those who age with a physical or intellectual disability acquired in early or mid-life. An increasing number of individuals fall into this category. The life expectancy of people with disability onset in early and mid-life has increased dramatically, at a more rapid rate than that of the general population as discussed at GOWD by Mattie in her presentation *New technologies for people aging with a disability*.

The aging with disability population may face unique circumstances in later life. These include: “accelerated aging, secondary conditions, post-onset syndromes, and different incidence rates of age-related conditions” [3]. The onset of secondary conditions occurs when the functional symptoms of aging are “superimposed on the primary disability” [4, p. 38]. This experience is related to that of premature or accelerated aging, where individuals with disabilities in early or mid-life can experience the symptoms of functional decline, which are typically associated with later life, 10 to 20 years earlier than their able-bodied counterparts [3, 5]. In her GOWD conference presentation, *Aging with an existing physical disability*, Lisa Iezzoni cited concerns among the aging with disability population around this worsening of existing disability or the development of other related conditions or complications as a result of their disability. She emphasized that the aging with disability trend requires recognition of variance in the combined overlay of the aging and disability experiences. Bridging aging and disability in areas of research, policy, and practice emerged as a prominent theme in GOWD conference proceedings in support of this endeavour.

## Focal area #1: the need to forward bridging between aging and disability sectors

As noted by Bickenbach et al. [6] the need to bridge aging and disability is an internationally pressing concern given current demographic trajectories, requiring movement beyond the traditional dichotomization of older and younger adults, aging with and aging into disability. Presenters at GOWD consistently discussed the need to build bridges across aging and disability sectors. In her presentation titled, *Trends in health care reform*, Leonardi (2011) described the two fields of research, policy, and practice as operating as ‘discrete realities’ historically viewed by public policies and funding mechanisms as separate entities. She stated that the distinct conceptualizations of aging and disability hinder opportunities for collaborative efforts between their respective constituencies. As Big by pointed out during her presentation titled, *Key Issues and research priorities affecting social outcomes for an older population: differentiating adulthood, hearing and acting on the perspectives of older people with intellectual disability*, this is in spite of the fact that the policy agendas of aging and disability constituencies are similar in focus, with both groups emphasizing such priorities as “rights, participation, choice and inclusion”. In *The best national programs protecting the rights of persons aging with disabilities,* Bickenbach identified this as a “strangely absent alliance” because of the clear strategic value in joining efforts to advocate based on human rights.

Prior scholarship has pointed out that older and younger persons with disabilities are often viewed and dealt with as entirely different constituency and needs groups, receiving different entitlements to care and support services and therefore making joint advocacy efforts across aging and disability groups complex [7, 8]. Bishop and Hobson’s presentation, *Aging with an adult-onset physical disability: a scoping review* confirmed this segmentation with scholarly literature as well. Their findings showed limited overlap between models of aging and disability and discipline-specific distinctions in the language used to discuss disability between aging and disability research. Despite the tendency to bifurcate aging from disability, Salvador-Carulla’s, in *Bridging aging and disability networks: strategies for translating knowledge into practice,* highlighted the significant number of people living with the reality of both experiences—that people with disabilities are aging and that a high number of people acquire disabilities as they age—as a case for bridging the gaps between aging and disability sectors. Multiple GOWD presenters indicated that aging and disability are a continuum with people across the age span facing similar issues and concerns. As McDaid discussed at GOWD in *Future research agenda: making the economic case for investing in support for growing older with disabilities*, many existing models concentrate on the economic cost of disability and aging, while giving little attention to the benefits and links between the two. He cites a growing need to know more about the positive effects of aging and disability and making an economic case for investing in supports for persons growing older with disabilities.

After the GOWD meetings, bridging was defined by the expert panel of meeting participants in the Toronto Declaration with the aim of providing a context for future work on bridging aging and disability:

Bridging encompasses a range of concepts, tasks, technologies and practices aimed at improving knowledge sharing and collaboration across stakeholders, organizations and fields in care and support for persons with disabilities, their families, and the aging population. Bridging tasks include activities of dissemination, coordination, assessment, empowerment, service delivery, management, financing and policy. The overall purpose of bridging is to improve efficiency, equity of care, inclusion and support at all levels, from the person to the society [9].

## Focal area #2: theoretical frameworks of individual aging that facilitate bridging

Theoretical frameworks for viewing individual aging emerged as important elements for facilitating bridging between the fields of aging and disability at the GOWD conference. Two primary models presenters discussed were the life course perspective on aging and a bio-psychosocial model of disability. Both theories support extending the conceptualizations of age and disability beyond that of life years lived and loss in function. Summaries of each are presented below.

### The life course perspective

The life course perspective proposes a holistic conceptualization of the human experience. It recognizes that age and disability are not defining traits of an individual, but overlapping phenomena that occur throughout the span of the life course [10, 11]. Inher FICCDAT presentation*, Key Issues and research priorities affecting social outcomes for an older population: differentiating adulthood, hearing and acting on the perspectives of older people with intellectual disability,* Christine Big by stated that the separation between the disability and aging sectors—particularly in the delivery of services—has led to a lack of awareness of the “complete picture of a person’s life”. Furthermore, she stated that there is no strong mandate in place to ‘know the whole person” or to consider a life course perspective. A lack of knowledge transfer and sharing to bridge gaps between these policy sectors means that there is a disconnect in current practices from understanding the lives and needs of people aging with or into disability.

A life course perspective encompasses five basic principles, as outlined by Elder et al. [12, p. 11–13]. These principles are:

1. *Life-span development*—understanding disability and aging experiences as lifelong processes.
2. *Agency*—the choices and actions made throughout the life course, influenced by the limitations of one’s personal circumstance, serve to shape their overall experience.
3. *Time and place*—the life course is shaped by historical, social, geographical contexts.
4. *Timing*—events and experiences, such as disability, impact individuals differently depending on *when* they occur.
5. *Linked lives*—emphasizes interdependence, “shared relationships” and the extent to which personal relationships and interactions can influence the lives of individuals and those they encounter.

This model shifts from age-specific chronological criteria toward prioritizing individual choice and circumstance and placing them within larger social and historical context [13]. It requires some recognition of the unique history of those aging with disabilities that may span over the course of their lifetime, something that may inform their service needs and experiences in later life [11]. This lens has utility for recognizing the differing aging experiences of those with an existing long-term disability, compared to those who acquire disability as they age. On this point writes:

The life course perspective of sociology offers a promising framework for expanding our knowledge of age-related changes associated with long-term disabilities. Unlike most rehabilitation approaches, which emphasize impairment and functional limitations, the life course perspective focuses on describing the temporal structure of disability and examining the consequences of variations in the timing of disability events for the well-being of survivors as they age. (p. 1) [5].

The above statement illustrates the extent to which a life course perspective is important to capturing the unique needs of people aging with disabilities.

### Bio-psychosocial model

GOWD presenters also drew on the bio-psychosocial perspective, a framework that integrates the dominant medical and social models of disability [14]. Here, disability is deemed to be a product of “biological, personal, and social forces” [15, p. 727]. It represents a movement away from thinking about disability from an entirely functional perspective, promoting the view that disability is multi-faceted, influenced by a number of factors that shape the experience. At GOWD, Leonard suggested in her presentatation,* Trends in health care reform, *that re-thinking disability will lead to “re-thinking the sense of politics itself” [10]. She supported a bio-psychosocial model of health as one that offers a multifaceted perception of disability, encompassing both health status and the physical and social environment. A holistic theoretical conceptualization of disability, inclusive of the environment, social and human factors is important because it promotes the notion that this is a condition spanning across all populations, placing “human beings as the base for policy and policy development” [10]. This conceptualization is based upon the definition of disability proposed by the World Health Organization (WHO), under its International Classification of Functioning (ICF). The ICF’s classification system has the potential to bridge understandings of disability. Although the success and global use of this model still needs to be measured and assessed [13], Leonardi et al. argue that supporting such a definition brings society closer to achieving “equal rights, opportunities, and participation” for people with disabilities [16].

This multi-layer conception may also help to bridge knowledge gaps in the intersection of disability with the aging process in a way that more limited, and dominant functional or biomedical, perspectives do not allow. Additionally, broadening the definition in this way may help to reduce discrimination or exclusion experienced by those aging with or into disability that result from deeply embedded cultural and social norms held by the larger society. In his GOWD conference presentation,* Ableism: a theoretical framework to evaluate how we expect people to age*, Wolbring raises the issue of discrimination and ableism as particularly salient in relation to the paradigm shift that is currently occurring in health care delivery towards patient-driven care, where patients increasingly desire control over their own health due to the growing number of health service technologies available to them. He notes the relevance of understanding issues such as patient-driven care within a bio-psycho-social framework that considers ramifications of ableism on the self-determination of individuals within health care systems.

## Focal area #3: bridging through consumer participation and involvement

At the GOWD conference, a consensus emerged that research, practice and policy is truly effective only if the people that it concerns are directly involved. Those aging with and into disabilities must therefore be directly involved as key actors. Iezzoni highlighted this in her presentation, *Aging with an existing physical disability*, stating that one of the most important things researchers and policy-makers could do is to not make assumptions about the needs, abilities and experiences of people aging with disability [8]. Instead, she indicated that they should consult individuals living with disability and their families and work with them in partnership to achieve identified goals related to positive aging. Big byechoed this call indicating that older adults aging with intellectual disabilities are particularly vulnerable to larger social biases and assumptions. She stated, however, that persons aging with intellectual and developmental disabilities have strong ideas about the future direction of their own lives and should be given the opportunity to participate in the decision-making process in her presentation, *Key Issues and research priorities affecting social outcomes for an older population: differentiating adulthood, hearing and acting on the perspectives of older people with intellectual disability*.

Such an approach endorses a participatory action approach, where professionals work in partnership with members of the community so that ‘stakeholders’ with a direct interest in the resulting practice and policy have a voice [17]. In his conference presentation entitled *Growing older with a disability in Asia*—*bridging policies and practices*, Mendes spoke of the need to develop and strengthen such partnerships, particularly in Asian nations. He discussed bridging aging and disability policies and practices, noting the importance of empowering persons with disabilities to be active participants in the political process. This includes changing policy goals and involving a number of actors—including aging organizations and other NGOs—so that policy-making authority is spread amongst multiple sectors rather than being the sole responsibility of the state. Sunsern, Pothong, and Rukkaumsookechoed this need as they presented findings on a study exploring needs of people with disabilities of all ages in a single province in Thailand in the presentation titled, *Exploring Thai community support for disabled people*. Results of their qualitative work indicated that collaboration across professional fields of practice and with people with disabilities and their families is seen as crucial to meeting the community-based needs of all persons with disabilities.

Examples of the importance of engaging individuals with disabilities and their families as collaborators in addressing health and well-being concerns at GOWD included a presentation by McWilliam, Forbes, and Forchuk titled, *Family caregivers’ experience of in-home knowledge translation*, describing the challenges of working with caregivers to implement an evidence-based approach to at-home management of urinary incontinence for older adults. Findings supported the need for a dynamic partnership between caregivers and professional service providers to address caregiver questions and concerns and to ensure affinity to and success of the intervention. A different example of collaboration was provided by GOWD presenters Hughes and Cammack who described the web-based Tyze social network in their presentation, *Tyze: care, connect, contribute*. Tyze connects consumers, family members and professionals in a private, personalized care network with an interface that permits ongoing dialogue and connection among an individual’s formal and informal care network.

## Focal area #4: bridging through knowledge transfer

Knowledge transfer involves the movement of ideas, information and concepts between multiple sectors, including research and academic institutions, industries, policy-makers and the public [18]. McDaid et al. state that the transfer of knowledge is non-linear, requiring “active dialogue and exchanges between researchers, policy-makers, practitioners and client groups” [7]. They find that the sharing and exchange of knowledge establishes a stronger connection between research, policy and practice for “the public, civil society and private sectors, as well as across disciplines”. The Canadian Institutes of Health Research [19] further elaborates on the process of knowledge translation defining it as “the exchange, synthesis and ethically-sound application of knowledge—within a complex system of interactions among researchers and users—to accelerate the capture of the benefits of research for Canadians through improved health, more effective services and products, and a strengthened health care system.” The process of knowledge translation has been defined in the health sector as “the synthesis, exchange, and application of knowledge by relevant stakeholders to accelerate the benefits of global and local innovation in strengthening health systems and improving people’s health” [20].

At GOWD, Salvador-Carulla pointed out that effective knowledge transfer is a critical foundation for bridge building across aging and disability that is required if the commonality of needs and outcomes are to be effectively presented and addressed in his presentation,* Bridging aging and disability networks: strategies for translating knowledge into practice*. He further stated that the implications of the current separation between the fields of aging and disability include missed opportunities for “knowledge, innovation and policy change”. Campbell [21] claims that needed results and knowledge translation require “researchers, practitioners, and advocates in the aging and disability nexus [to] adopt a new mind-set, learn a new vocabulary, and apply new tools” (p. 230). Rather than adapting the principles of one group to the other, a new set of principles must be adopted in the translation and sharing of knowledge.

## Focal area #5: bridging opportunities in long-term supports and services and assistive technologies

At GOWD, a substantial number of presentations related to the topic of long-term supports and services including assistive technology. As discussed at GOWD by both Iezzoni in her presentation, *Aging with an existing physical disability* and Mattie in her presentation, *New technologies for people aging with a disability*, a number of social, socioeconomic, and health related challenges emerge with the combined experiences of aging and disability that influence support and service needs, including limited financial resources or fixed income, widowhood, home access, aging family caregivers, and limited access to public transportation. Currently, categorical requirements—such as age or disability type—typically govern the allocation of services and supports. People aging with long-term disabilities pose a particular challenge within this dynamic because they do not necessarily fit within one service network or the other, but often both.

Acknowledged by Campbell and other organizers of the GOWD meeting (personal communication, July 26, 2011), there is a lack of evidence-based practices in the most efficient delivery mechanisms of support services for this particular population, and while a number of evidence-based and practice guidelines have been developed, their efficacy has not been successfully tested on those aging with disabilities. According to Big by, service providers are often unable to translate and adapt the principles utilized for younger people with disabilities to those who are aging as noted in her presentation, *Key Issues and research priorities affecting social outcomes for an older population: differentiating adulthood, hearing and acting on the perspectives of older people with intellectual disability*. In their presentation at the GOWD conference, Moll and Cott (2011) discussed the phenomenon of growing older with a disability and the current gap in services to accommodate this group in both health care and rehabilitative services in *Growing up–growing older with a physical impairment: the paradox of normalization*. Their findings indicate that while rehabilitation is provided to people with disabilities in youth, the health care system is not equipped to aid individuals with childhood onset impairments in later years of life. Those who experience premature aging or secondary conditions therefore often lack access to the appropriate rehabilitation services, yet with advancements in medicine and technology the life expectancy of these individuals has increased [37]. Strategies that foster development of new knowledge and effective transfer of existing knowledge can help improve supports and services for persons aging with disabilities.

Several GOWD presenters provided specific examples of bridging in the area of long-term supports and services. Sherwood, Kinney, and Franck’s (2011) presentation, *Physical activity and nutrition for adults aging with multiple disabilities*, describes a fourteen-week fitness program designed for adults with a single physical or cognitive disability and piloted with persons aging with multiple disabilities. Slight modification in program design permitted the continual adaptation and evaluation of the program model to fit this group. Heller, Arnold, van Heumen, McBride, and Factor’s (2011) presentation, *Consumer-directed support: impact of hiring practices on adults with ID/DD and families*, reported the outcomes of application of a consumer-directed long-term supports and services model that has been used across younger and older adult populations in the US. Heller and colleagues found that hiring of family members and friends did not seem to restrict the self-determination of persons with disabilities within the consumer-direction model thus addressing concerns about using a consumer-direction model across disability populations. In the presentation *Spinal Cord Connections Resource Centre: promoting health for people growing older with a spinal cord injury,* Millsand Doyle (2011) presented a program of specialized resource centers in Ontario, Canada that provides education on strategies for healthy aging to people with spinal cord injuries. Other presenters, like Reed who presented *The hard of hearing club: a social model of hearing rehabilitation for seniors* and Bridge who discussed *Age-specific housing in Australia for low to moderate income older people* highlighted intervention needs of older adults experiencing disabilities that also align with concerns of younger persons with disabilities relating to access and availability of effective social care supports.

GOWD presenters also focused on the bridging potential of assistive and information technologies. The use of assistive technology and information technology has increasingly become an important part of health and social care [37]. Thoreau presented *Mobility scooter use and the physical functioning of older adults*, an examination of physical and cognitive health markers over time between older adult technology and non-technology users. The presentation highlighted the universality of this study design for investigating this issue among younger adults with disabilities as well. In a related, but contrasting presentation, *Opportunity for meaningful occupation through powered mobility in old age,* Nilsson and Wang suggested the importance of powered mobility in improving access and autonomy of persons aging with and into disability. Finally, Mattie highlighted the importance of understanding the specific needs of those aging into and with disabilities so that devices and technologies can be adapted in a way that is beneficial and usable to these growing populations in her presentation, *New technologies for people aging with a disability.* The use of such devices allows individuals to lead a more inclusive lifestyle by minimizing the effects of disability that result from physical barriers in society [22].

At the policy level, two notable examples of bridging in the sector of long-term supports and services were presented. At GOWD, Leonard is poke of the COURAGE in EUROPE project as one such endeavorin her presentation, *Trends in health care reform*. It is a three-year effort, which aims to develop a measure of health and health-related outcomes, for an ageing population. The end result will measure these effects and produce a database to better shape a number of different policy arenas, including the health, social and economic spheres. The gain of such a model is better understanding of the health and service needs of aging populations and the production of data usable by consumers, industries, and policy-makers alike. In her GOWD presentation entitled *Aging and disability bridging experiments in the U.S.: progress and challenges*, Putnam (2011) discussed recent bridging efforts and identified several bridging initiatives and opportunities. Putnam offered that U.S. government data does indicate some success in programs designed for persons with disabilities of any age to obtain community-based services. However she indicated that eligibility requirements remain varied between support services and care benefits for both the aging and disability sectors. Putnam stated that additional opportunities to explore bridging included training aging and disability professionals to support persons aging with disabilities.

## Conclusions

The number of people aging with or into disability is increasing dramatically worldwide. In this report, five focal areas from the GOWD meeting presentations were identified: 1) the need to forward bridging between aging and disability sectors, 2) theoretical frameworks of individual aging that facilitate bridging, 3) bridging through consumer participation and involvement, 4) bridging through knowledge transfer, and 5) bridging opportunities in long-term supports and services and assistive technologies. These focal areas provide an initial foundation for development of a bridging framework in aging and disability and serve as the foundation for the Toronto Declaration [9].
